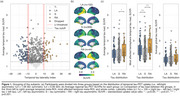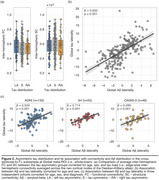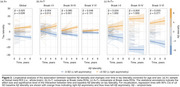# Hemispheric Asymmetry of Amyloid Deposition Influences Asymmetric Tau Pathology in Alzheimer’s Disease

**DOI:** 10.1002/alz70862_110048

**Published:** 2025-12-23

**Authors:** Toomas Erik Anijärv, Ruben Smith, Lyduine E. Collij, Harry H Behjat, Alexa Pichet Binette, Jonathan Rittmo, Linda Karlsson, Olof Strandberg, Danielle van Westen, Jacob W. Vogel, Erik Stomrud, Sebastian Palmqvist, Niklas Mattsson‐Carlgren, Rik Ossenkoppele, Nicola Spotorno, Oskar Hansson

**Affiliations:** ^1^ Clinical Memory Research Unit, Lund University, Lund Sweden; ^2^ Clinical Memory Research Unit, Lund University, Malmö, Skåne Sweden; ^3^ Memory Clinic, Skåne University Hospital, Malmö, Skåne Sweden; ^4^ Radiology and Nuclear Medicine, Vrije Universiteit Amsterdam, Amsterdam UMC, Amsterdam Netherlands; ^5^ Clinical Memory Research Unit, Department of Clinical Sciences Malmö, Faculty of Medicine, Lund University, Lund Sweden; ^6^ Amsterdam Neuroscience, Brain Imaging, Amsterdam Netherlands; ^7^ Department of Physiology and Pharmacology, Université de Montréal, Montréal, QC Canada; ^8^ Centre de recherche de l'institut universitaire de gériatrie de Montréal (CRIUGM), Montréal, QC Canada; ^9^ Department of Clinical Sciences Malmö, SciLifeLab, Lund University, Lund Sweden; ^10^ Clinical Memory Research Unit, Department of Clinical Sciences, Lund University, Lund Sweden; ^11^ Diagnostic Radiology, Institute for Clinical Sciences Lund, Lund University, Lund Sweden; ^12^ Department of Clinical Sciences Malmö, Faculty of Medicine, SciLifeLab, Lund University, Lund Sweden; ^13^ Department of Neurology, Skåne University Hospital, Lund Sweden; ^14^ Wallenberg Center for Molecular Medicine, Lund University, Lund Sweden; ^15^ Amsterdam Neuroscience, Neurodegeneration., Amsterdam Netherlands; ^16^ Alzheimer Center Amsterdam, Neurology, Vrije Universiteit Amsterdam, Amsterdam UMC location VUmc, Amsterdam Netherlands; ^17^ Clinical Memory Research Unit, Department of Clinical Sciences Malmö, Lund University, Lund Sweden

## Abstract

**Background:**

Tau pathology distribution in Alzheimer’s disease (AD) shows individual spatial heterogeneity, including hemispheric asymmetry. The mechanisms underlying this asymmetry remain unclear. This study explored whether tau asymmetry is linked to reduced inter‐hemispheric connectivity, potentially restricting tau spread, or reflects asymmetry in amyloid‐beta (Aβ) distribution, indicating hemisphere‐specific vulnerability to early Aβ pathology.

**Method:**

The study included 837 Aβ‐positive (CSF Aβ42/40<0.08 or cortical Aβ‐PET>1.033) participants from the Swedish BioFINDER‐2 cohort with available tau‐PET scan(s). A cross‐sectional subsample of 452 subjects with evidence of tau pathology based on temporal meta‐ROI (Braak I‐IV; SUVR>1.362) was selected and categorised as left asymmetric (*n* = 102), symmetric (*n* = 306), or right asymmetric (*n* = 44) using a laterality index (LI; see Figure 1). First, edge‐wise inter‐hemispheric structural (diffusion‐MRI, *n* = 352) and functional (rs‐fMRI, *n* = 318) connectivity were compared between the tau asymmetry groups using the Desikan‐Killiany parcellation. Only the top 10% inter‐hemispheric connections identified in a separate control sample were analysed. Second, the association between Aβ and tau laterality patterns was investigated using linear regression on LI values and validated in three independent cohorts (OASIS‐3, A4, ADNI). Finally, in a longitudinal subsample (*n* = 289; average follow‐up = 2.9 years) of Aβ‐positive participants with multiple tau‐PET scans, stratified into A+T‐ (*n* = 180) and A+T+ (*n* = 109) groups based on pathology at baseline, linear mixed effect models were used to assess the association between baseline Aβ laterality and tau laterality change over time.

**Result:**

Cross‐sectionally, no differences in average edge‐wise inter‐hemispheric functional or structural connectivity were found between tau asymmetric and symmetric groups (Figure 2a). In contrast, a strong association was observed between tau and Aβ laterality patterns (Figure 2b; β=0.632, *p* <0.001), which was replicated in three independent cohorts (Figure 2c; all *p* <0.005). In the longitudinal A+ sample, the degree of Aβ asymmetry at baseline predicted progression of tau laterality over time (Figure 3a; β=0.025, *p* = 0.028), with the strongest interaction effect in the Braak III‐IV meta‐ROI in A+T‐ individuals (Figure 3b; β=0.080, *p* <0.001) but not in A+T+ (Figure 3c).

**Conclusion:**

These findings suggest that tau asymmetry is not associated with differences in macro‐scale inter‐hemispheric individual connectivity but reflects hemispheric differences in vulnerability to Aβ pathology.